# Incidence of type 2 diabetes before and during the COVID-19 pandemic in Naples, Italy: a longitudinal cohort study

**DOI:** 10.1016/j.eclinm.2023.102345

**Published:** 2023-12-05

**Authors:** Raffaele Izzo, Daniela Pacella, Valentina Trimarco, Maria Virginia Manzi, Angela Lombardi, Roberto Piccinocchi, Paola Gallo, Giovanni Esposito, Maria Lembo, Gaetano Piccinocchi, Carmine Morisco, Gaetano Santulli, Bruno Trimarco

**Affiliations:** a Department of Advanced Biomedical Sciences, “*Federico II*” University, Naples, Italy; b Department of Public Health, “*Federico II*” University, Naples, Italy; c Department of Neuroscience, Reproductive Sciences, and Dentistry, “*Federico II*” University, Naples, Italy; d Department of Microbiology and Immunology, Fleischer Institute for Diabetes and Metabolism (*FIDAM*), Albert Einstein College of Medicine, New York City, NY, USA; e “*Luigi Vanvitelli*” Hospital, Naples, Italy; f *COMEGEN* Primary Care Physicians Cooperative, Italian Society of General Medicine (*SIMG*), Naples, Italy; g International Translational Research and Medical Education (*ITME*) Consortium, Academic Research Unit, Naples, Italy; h Italian Society for Cardiovascular Prevention (*SIPREC*), Rome, Italy; i Department of Medicine, Wilf Family Cardiovascular Research Institute, Einstein-Mount Sinai Diabetes Research Center (*ES-DRC*), Albert Einstein College of Medicine, New York City, NY, USA; j Department of Molecular Pharmacology, Einstein Institute for Aging Research, Institute for Neuroimmunology and Inflammation (INI), Albert Einstein College of Medicine, New York City, NY, USA

**Keywords:** COVID-19, Diabetes mellitus, Public health, SARS-CoV-2, T2DM

## Abstract

**Background:**

The association of COVID-19 with the development of new-onset diabetes has been recently investigated by several groups, yielding controversial results. Population studies currently available in the literature are mostly focused on type 1 diabetes (T1D), comparing patients with a SARS-CoV-2 positive test to individuals without COVID-19, especially in paediatric populations. In this study, we sought to determine the incidence of type 2 diabetes (T2D) before and during the COVID-19 pandemic.

**Methods:**

In this longitudinal cohort study, we analysed a cohort followed up over a 6-year period using an Interrupted Time Series approach, *i.e.* 3-years before and 3-years during the COVID-19 pandemic. We analysed data obtained from >200,000 adults in Naples (Italy) from January 1st 2017 to December 31st 2022. In this manner, we had the opportunity to compare the incidence of newly diagnosed T2D before (2017–2019) and during (2020–2022) the COVID-19 pandemic. The key inclusion criteria were age >18-year-old and data availability for the period of observation; patients with a diagnosis of diabetes obtained before 2017 were excluded. The main outcome of the study was the new diagnosis of T2D, as defined by the International Classification of Diseases 10 (ICD-X), including prescription of antidiabetic therapies for more than 30 days.

**Findings:**

A total of 234,956 subjects were followed-up for at least 3-years before or 3-years during the COVID-19 pandemic and were included in the study; among these, 216,498 were analysed in the pre-pandemic years and 216,422 in the pandemic years. The incidence rate of T2D was 4.85 (95% CI, 4.68–5.02) per 1000 person-years in the period 2017–2019, *vs* 12.21 (95% CI, 11.94–12.48) per 1000 person-years in 2020–2022, with an increase of about twice and a half. Moreover, the doubling time of the number of new diagnoses of T2D estimated by unadjusted Poisson model was 97.12 (95% CI, 40.51–153.75) months in the prepandemic period *vs* 23.13 (95% CI, 16.02–41.59) months during the COVID-19 pandemic. Interestingly, these findings were also confirmed when examining patients with prediabetes.

**Interpretation:**

Our data from this 6-year study on more than 200,000 adult participants indicate that the incidence of T2D was significantly higher during the pandemic compared to the pre-COVID-19 phase. As a consequence, the epidemiology of the disease may change in terms of rates of outcomes as well as public health costs. COVID-19 survivors, especially patients with prediabetes, may require specific clinical programs to prevent T2D.

**Funding:**

The US National Institutes of Health (NIH: NIDDK, NHLBI, NCATS), 10.13039/100001583Diabetes Action Research and Education Foundation, Weill-Caulier and Hirschl Trusts.

**Research in context**

**Evidence before this study**

We searched PubMed on July 18 2023 for English language publications using a combination of the search terms "COVID-19", "new-onset type 2 diabetes", and "adults", retrieving no publications. Reports on diabetes and COVID-19, limited in number and in the size of the studied groups, are focused on T1D and/or pediatric populations.

**Added value of this study**

In our study, analyzing a large real-world dataset of adult subjects, the incidence rate of newly diagnosed T2D was 4.85 per 1000 person-years in the period 2017–2019 (before the COVID-19 pandemic), *vs* 12.21 per 1000 person-years in 2020–2022. The estimated doubling time of the number of new T2D diagnoses was 97.12 months in the prepandemic period *vs* 23.13 months during the COVID-19 pandemic. These statistically significant differences were also confirmed in a cohort of individuals with prediabetes.

**Implications of all the available evidence**

No previous study, to the best of our knowledge, has compared the incidence of T2D in the same population followed for 3 years before and during the pandemic. Collectively, our data clearly indicate that COVID-19 is associated with an increased incidence of T2D. Further research is needed to understand the exact mechanisms underlying the association of COVID-19 and new-onset T2D.

## Introduction

On May 4th 2023, the World Health Organization has declared that COVID-19 pandemic is over. However, it is now clear that there are numerous sequelae of this pandemic that the Health Systems of all Countries have still to face. Among these post-acute conditions, one of the most relevant is the increase in incident diabetes mellitus.

New cases of diabetes have been widely reported during the acute phase of COVID-19^1^, ^2^, ^3^, ^4^, ^5^, ^6^, ^7^, ^8^, ^9^, ^10^, ^11^, ^12^, ^13^, ^14^, ^15^, ^16^, ^17^, ^18^, ^19^, ^20^, ^21^, ^22^, ^23^, ^24^, ^25^, ^26^, ^27^ and Xie and Al-Aly^28^ specifically demonstrated, in a cohort built using electronic health records from the U.S. Department of Veterans Affairs, that over a median follow-up period of 12 months the rate of incident diabetes was 46% higher in people diagnosed with COVID-19 than in those without COVID-19. More recently, Naveed and coworkers^29^ have conducted a population-based cohort study, using the British Columbia COVID-19 Cohort, a surveillance platform that integrates COVID-19 data with administrative data sets by comparing new cases of diabetes in individuals tested for SARS-CoV-2 in 2020 and 2021, with those of sex- and age-matched individuals who tested negative, at a 1:4 ratio. They found that incident diabetes rate was significantly higher in the exposed *vs* nonexposed group, showing that the risk was actually increasing with the severity of the disease, concluding that SARS-CoV-2 infection was associated with a higher risk of diabetes and may have contributed to a 3%–5% excess burden of diabetes.^29^ To further clarify these aspects, Zhang and colleagues^27^ performed a systematic review and meta-analysis of cohort studies assessing new-onset diabetes after COVID-19. At all ages, there was a statistically significant positive association between infection with COVID-19 and the risk of diabetes: <18 years: RR = 1.72 (1.19–2.49), ≥18 years: RR = 1.63 (1.26–2.11), and >65 years: RR = 1.68 (1.22–2.30). The relative risk of diabetes in different gender groups was about 2 (males: RR = 2.08 [1.27–3.40]; females: RR = 1.99 [1.47–2.80]).

These findings provide strong support for a diabetogenic effect of COVID-19, beyond the well-recognized stress response associated with severe illness,^30^, ^31^, ^32^, ^33^, ^34^, ^35^ and may also help identifying the exact mechanisms underlying the persistence of metabolic alterations observed in long-COVID.^36^, ^37^, ^38^, ^39^, ^40^ On the other hand, other reports did not detect any significant association between COVID-19 and new-onset diabetes.^41^, ^42^, ^43^, ^44^ Notably, the data currently available in the literature merely refer to comparisons between groups of patients who certainly had a positive SARS-Cov-2 test *vs* individuals with similar demographic characteristics who did not have COVID-19, thus assessing the effect of the long-term individual COVID-19 infection (post-acute sequelae) on the incidence of diabetes.^3^ However, it should be considered that the attenuation of the severity of COVID-19 symptoms and the possibility of autonomously performing less-sensitive tests has resulted in some COVID-19 infections not being diagnosed, making the evaluation of this complex phenomenon, and consequently its aftermaths on the public health organizations of different countries, less reliable.

In the present study we specifically analysed the impact of the pandemic in a broader force on the incidence of diagnoses of new-onset type 2 diabetes (T2D) in individuals residing in the city of Naples in Southern Italy, followed by their primary care physicians from 2017 to 2022. Specifically, we performed a single cohort study assessing the incidence of T2D using an interrupted time series (ITS) approach: a three-year period before the pandemic (*i.e.* 2017–2019) and a similar period of time during the pandemic (*i.e.* 2020–2022).

## Methods

### Study design

In this longitudinal cohort study, we harnessed data obtained from COMEGEN (“*COoperativa di MEdicinaGENerale*”: General Medicine Cooperative), a cooperative of primary physicians in the Naples Local Health Authority of the Italian Ministry of Health (ASL Napoli 1 Centro).^45^ Founded in 1997, COMEGEN today includes 140 physicians who are all connected in a network and employ the same computerized medical record software, creating a unique database containing the medical records of more than 200,000 adult patients. Medical records are updated daily by each physician, who uploads all data of her/his outpatient activity. The territorial distribution of those assisted by these doctors is similar to that of the city population recorded by the National Institute of Statistics (ISTAT), with no differences in terms of geographic area or aggregation by age.^45^^,^^46^ The COMEGEN database collects diagnoses according to the International Classification of Diseases 10 (ICD-X) recorded by primary care providers, also using standardized codes for all prescribed diagnostic assessments. The main outcome of the study was the new diagnosis of T2D assessed by the ICD-X codes E11 (“T2D”) and E13X (“Other specified diabetes mellitus”) with prescription of antidiabetic therapies for more than 30 days. Diagnoses of T1D, including the specific code E08 were not included. Pharmaceutical prescriptions are recorded in the COMEGEN database with the date, commercial name and active ingredients, alongside the quantities and methods of administration. In addition, the database includes data on vital parameters, weight, height, BMI, waist circumference, chronic conditions, medical visits, hospitalizations, emergency department visits, prescription drug dispensations, testing and vaccinations (including for COVID-19), date and cause of death. All these precious pieces of information allow for real-time provision of data related to the management of patients in terms of processes and outcomes, use of drugs, diagnostic investigations, and the complexity and comorbidities of the assisted population.^46^ The evaluation of person-time is crucial for calculating incidence rates and the COMEGEN allows an accurate assessment of incidence rates in this sense, by specifying when each individual has been entered in the database and started contributing data to the cohort, as well as the dates of death, end of follow-up, and end of observation.

We collected data from January 1st 2017 to December 31st 2022. The COMEGEN database provided demographic and clinical data. Laboratory measurements and medication data were available, as well. Information on COVID-19 was obtained from the Campania Region COVID-19 shared data resource.

This cohort study followed the ‘Strengthening the Reporting of Observational Studies in Epidemiology’ (STROBE) reporting guidelines. The Ethics Board at the “ASL Napoli 1 Centro” reviewed and approved this study and granted a waiver of informed consent.

The exposure was the beginning of COVID-19 pandemic and the primary outcome was newly diagnosed T2D. At the beginning of each 3-year observation period we removed from the study cohort all individuals with a record of HbA1c >6.5% (48 mmol/mol), or a previous diagnosis of diabetes as defined by the ICD-10 codes (E08.X to E13.X), or prescription record of anti-diabetic medications for more than 30 days.

We assessed the changes in T2D incidence using an ITS approach with the same cohort followed for 3 years before and 3 years during the COVID-19 pandemic. Only individuals whose information about T2D history were entirely available for the 6 years and in which selected demographic and clinical data were available at least for the 3 years before (2017–2019) or the 3 years after the COVID-19 outbreak (2020–2022) were included in the study. For this reason, although individuals were able to join the cohort over time as they became eligible, they did so in only limited numbers.

#### Inclusion criteria

Age >18-year-old; availability of information on diabetes history for the 6 years; availability of clinical and demographic data at least for the 3 years before or the 3 years after the COVID-19 outbreak.

#### Exclusion criteria

Age <18-year-old; diagnosis of diabetes (both T2D or T1D) before 2017; missing information on diabetes history, clinical, or demographic data.

To adjust for the difference in baseline characteristics between groups, we used clinically available predefined variables, which were selected based on a previous report^28^ where were used to define diabetic risk score. Predefined baseline variables included age, sex, BMI, smoking status (current smoker, former smoker, or never smoke). Comorbidities such as cancer, cardiovascular disease, chronic obstructive pulmonary disease, hypertension, hypercholesterolemia, and hypertriglyceridemia were also included as predefined covariates. Additionally, we also adjusted for laboratory test results including glycemia, HBA1c, creatinine, GPT and GOT.

### Statistical analysis

Data were summarized using mean (and S.D.) for continuous variables and absolute frequency (and percentage) for categorical variables and 95% confidence intervals (C.I.) were reported alongside. Incidence of events was reported as rate per 1000 person-years. Incidence growth rate over time was assessed using multiple linear regression on log-incidence. Two separate log-linear models (Poisson regression models) were fitted to descriptively report the unadjusted incidence rate in the pre-pandemic years (January 1st 2017 up to December 31st 2019) *vs* the incidence rate in the pandemic years (January 1st 2020 up to December 31st 2022) assuming a linear relationship between the log-incidence rate and time. A single log-linear model was additionally fitted to describe the trend of the incidence rate in the overall period. The goodness-of-fit was assessed inspecting the residual plot and the qq plot to assess the normality of the residuals (Supplementary Figure S1). Additionally, the Poissonness plot was adopted to assess the model fit (Supplementary Figure S2). An ITS analysis on the complete cohort (January 2017 up to December 2022) was performed to investigate the potential change in incidence trend in the pandemic years using an Autoregressive Integrated Moving Average (ARIMA) model. The hypothesis was that exposure to COVID-19 (January 2020 up to December 2022) could lead to a significant change in the slope of the trend (ramp term) without significant short-term changes (step term) in comparison with the counterfactual trend. Stationarity was checked using the Augmented Dickey–Fuller test. Values of p (autoregressive order), d (degree of differencing) and q (moving average order) for the ARIMA model were selected comparing the models’ performances using the Akaike Information Criterion (AIC). The cumulative risk curve was constructed using the Kaplan–Meier method, as we described.^47^

Presence of multicollinearity among variables was tested using the generalized variance inflation factor (GVIF); if GVIF >5, the variable was excluded from the models. Sensitivity analyses were conducted to assess the impact of missing data [imputation (using multiple imputation) *vs* no imputation] and outliers (including or excluding outliers); thus, 11,944 (5.1%) individuals with inconsistent or incorrect data were excluded from the analysis. Analyses were performed using the statistical software R, version 4.3.0. A two-tailed p < 0.05 was considered significant for all analyses.

### Role of the funding source

The funder of the study had no role in study design, data collection, data analysis, data interpretation, or writing of the report. R.I., D.P., G.P., and B.T. had access to dataset; G.S. and B.T. had final responsibility for the decision to submit for publication. All authors vouch for the completeness and accuracy of the data.

## Results

**Fig. 1:**
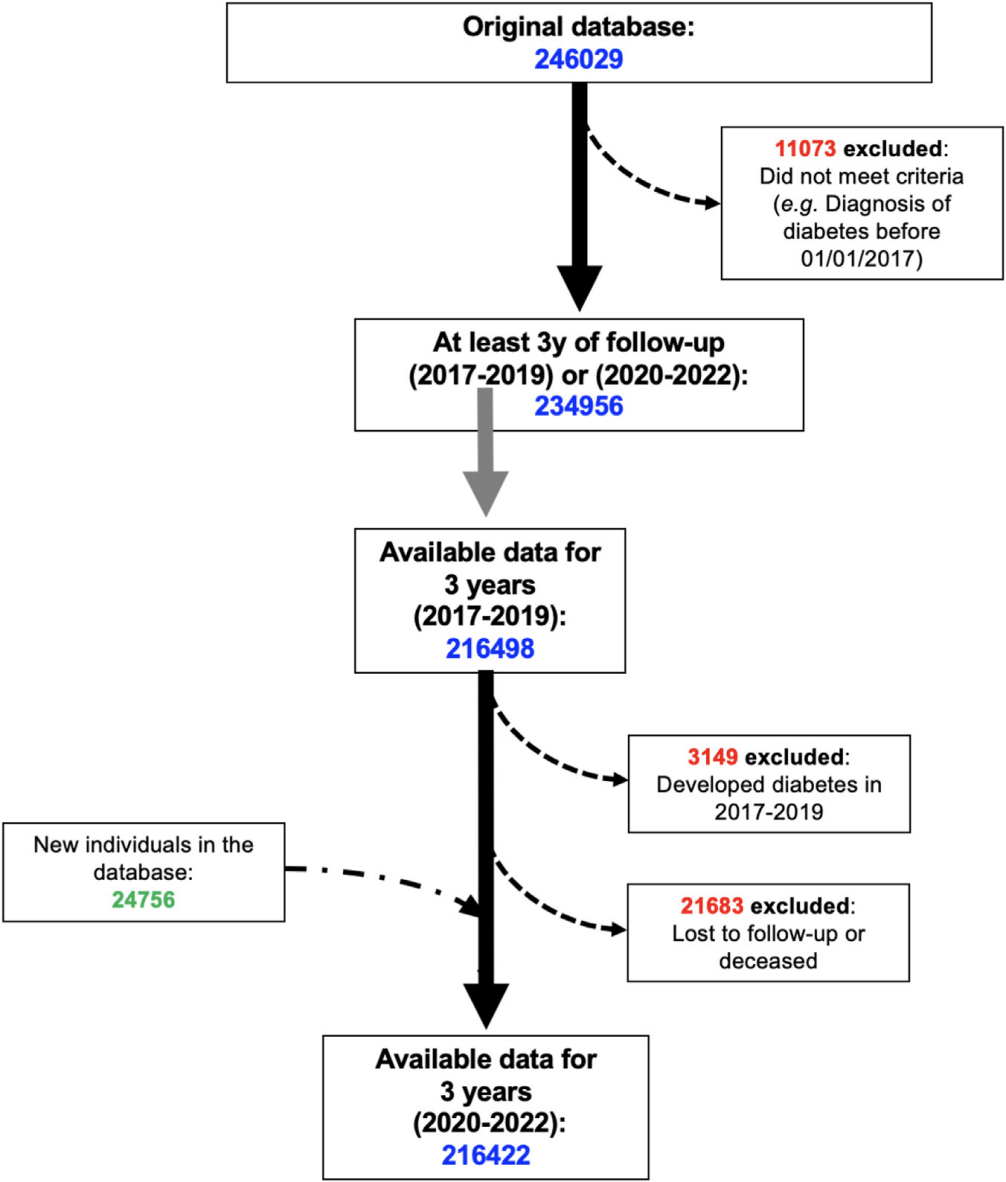
Flow chart of the study.

For the period 2017–2019, baseline data were available for 216,498 individuals (see flow chart in Fig. 1); out of the 216,422 individuals followed from January 1st 2020 to December 31st 2022, 30,517 had a positive test for COVID-19 between April 1st 2020 and December 31st 2022. The mean follow-up time was 5.79 ± 0.76 years (median follow-up time: 6 years). The main demographic and health characteristic of our study cohort are reported in Table 1.

**Table 1:** Demographic and clinical data divided from the 2017–2019 cohort and 2020–2022 cohort.

Variable	2017–2019 cohort (N = 216,498)	2020–2022 cohort (N = 216,422)
Age (years)	50 (33, 64)	53 (36, 68)
Female sex	117,188 (54%)	116,895 (54%)
Male sex	98,705 (46%)	98,926 (46%)
Smoking habit	20,490 (28%)	20,108 (28%)
BMI (kg/m^2^)	25.0 (22.7, 27.8)	25.0 (22.7, 27.8)
Creatinine (mg/dl)	0.86 (0.19)	0.86 (0.19)
Cancer (N)	43,027 (20%)	42,781 (20%)
COPD (N)	6145 (2.8%)	6304 (2.9%)
Hypertension (N)	66,006 (30%)	67,664 (31%)
Hypercholesterolemia (N)	25,280 (12%)	25,870 (12%)
Hypertriglyceridemia (N)	2442 (1.1%)	2465 (1.1%)
Glycemia (mg/dl)	92 (13)	92 (13)
HbA1c (mg/dl)	5.79 (0.67)	5.70 (0.60)
GPT (U/L)	20 (8)	20 (8)
GOT (U/L)	20 (6)	20 (6)
COVID-19 positive (N)	–	30,517 (14%)

Data are expressed as median (interquartile range, IQR), mean (standard deviation, SD), or number (percentage).

BMI: body mass index; COPD: chronic obstructive pulmonary disease; GOT: glutamic oxaloacetic transaminase; GPT: glutamic pyruvic transaminase; HbA1c: glycated haemoglobin.

**Fig. 2:**
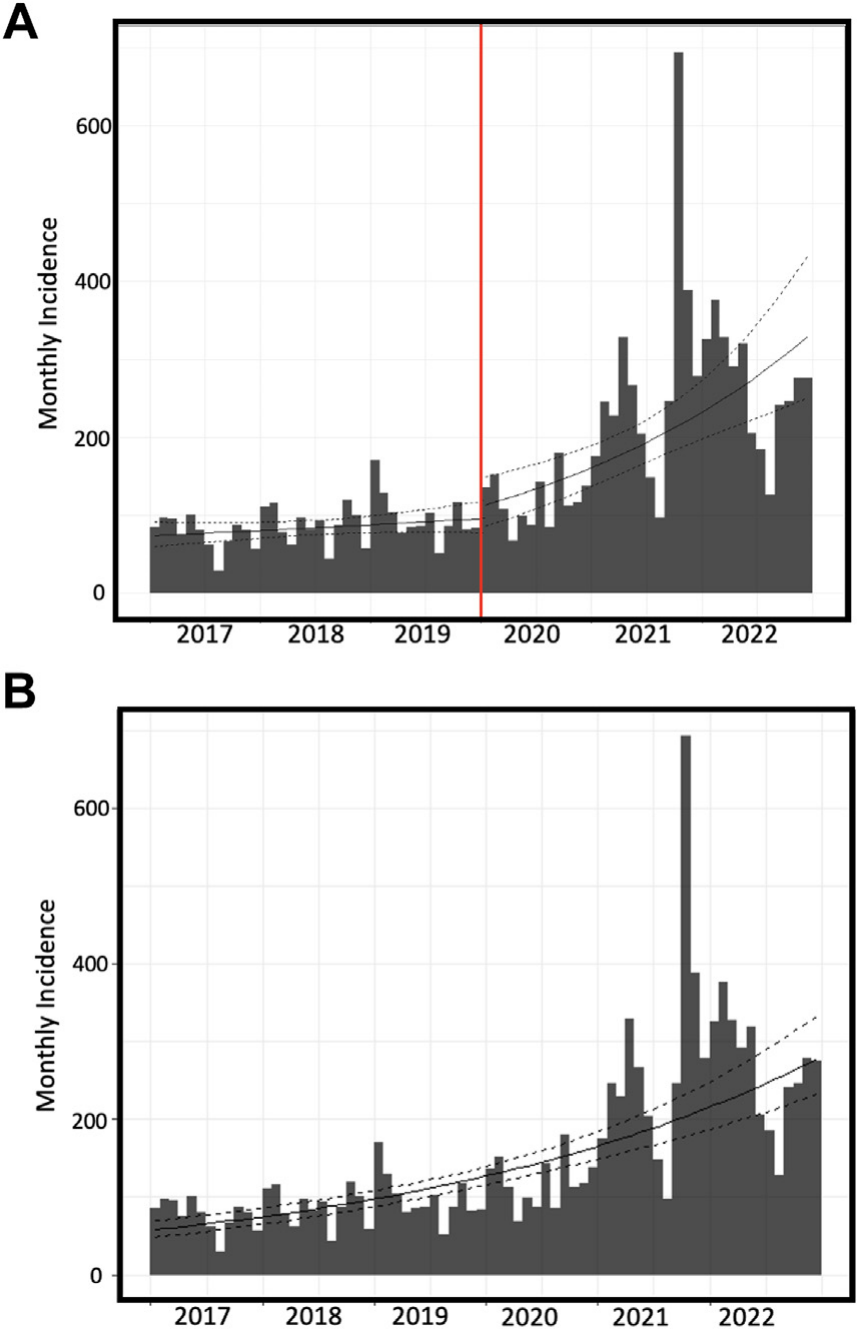
Interrupted time series (ITS) analysis of the monthly incidence of T2D in the 6-year observation period (2017–2022) fitted with two separate log-linear models, each on the two cohorts of individuals in the pre-pandemic and pandemic years (**A**). Monthly incidence of T2D in the 6-year observation period (2017–2022) estimated with the log-linear regression lines and 95% confidence bands on the whole cohort (**B**).

**Fig. 3:**
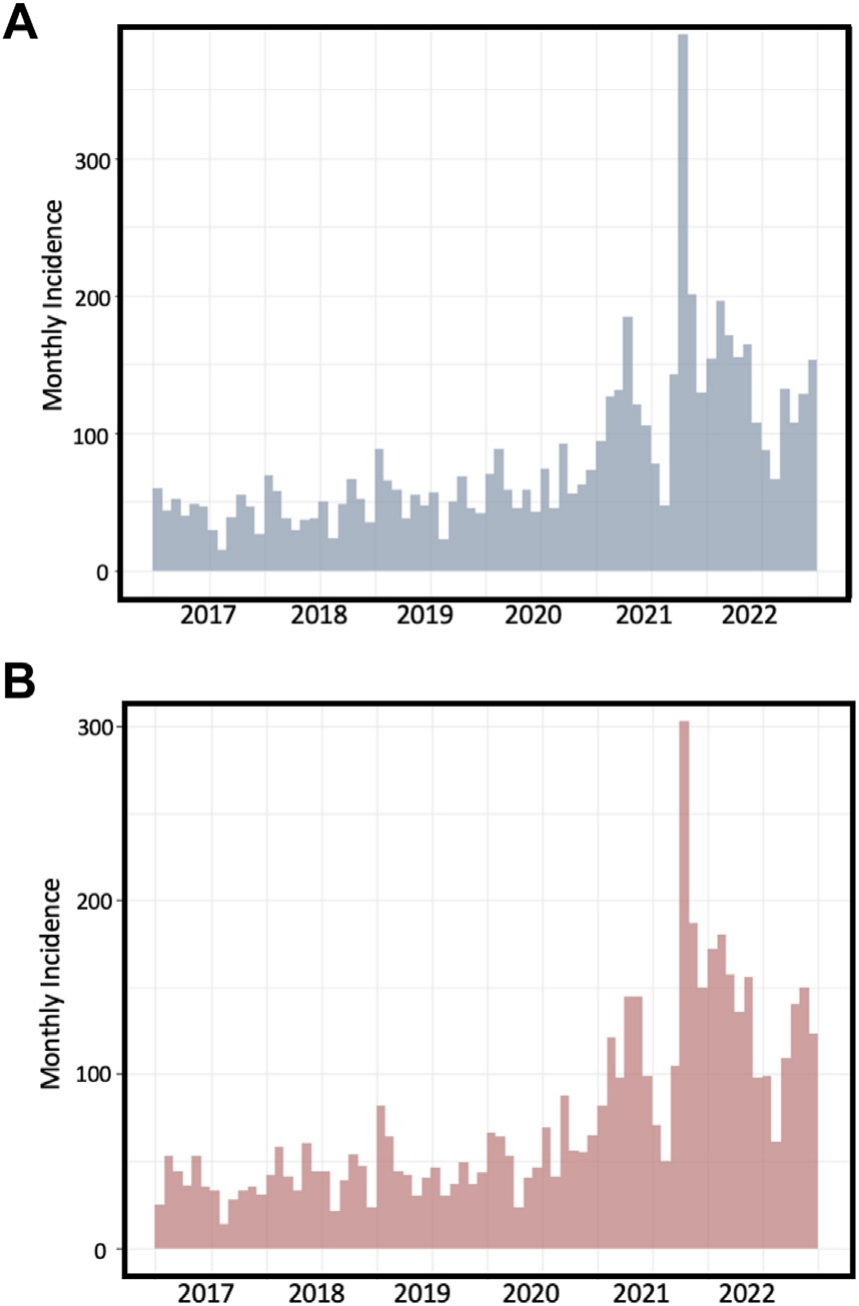
Monthly incidence of T2D in the 6-year observation period (2017–2022) in males (**A**) and in females (**B**).

The absolute numbers of new diagnoses of T2D before and during pandemic were 3149 and 7926, respectively. The T2D incidence rate was 4.85 (95% CI, 4.68–5.02) per 1000 person-years in the years 2017–2019 increasing to 12.21 (95% CI, 11.94–12.48) per 1000 person-years in the period 2020–2022, with an upsurge of about twice and a half (RR = 2.52). The log-linear models estimated separately for the two cohorts in each of the two 3-years periods indicated that the estimated monthly growth rate was approaching zero (0.007%, 95% CI, 0.000–0.017) in the pre-pandemic period while it was significantly increasing in the pandemic period (0.030%; 95% CI, 0.016–0.043, Fig. 2), in both males (Fig. 3A) and females (Fig. 3B). Strikingly, the estimated doubling time of the number of new diagnoses of T2D was 97.12 (95% CI, 40.51–153.75) months in the pre-pandemic period *vs* 23.13 (95% CI, 16.02–41.59) months in the pandemic period. The monthly growth rate estimated with the loglinear model for the overall 6-year period was 0.021% (95% CI, 0.017–0.026, Fig. 2).

ITS analysis conducted with ARIMA values (0,0,1) revealed, as hypothesised, an effect of the pandemic over time on the T2D incidence rate, with a significant change in the slope of the trend in the three pandemic years (“ramp” term b = 5.19, p = 0.023), while both the effect of pre-pandemic time and the effect of the beginning of the pandemic period (“step term”) were not significant. The plot in Supplementary Figure S3 displays the observed and the counterfactual regression lines.

**Fig. 4:**
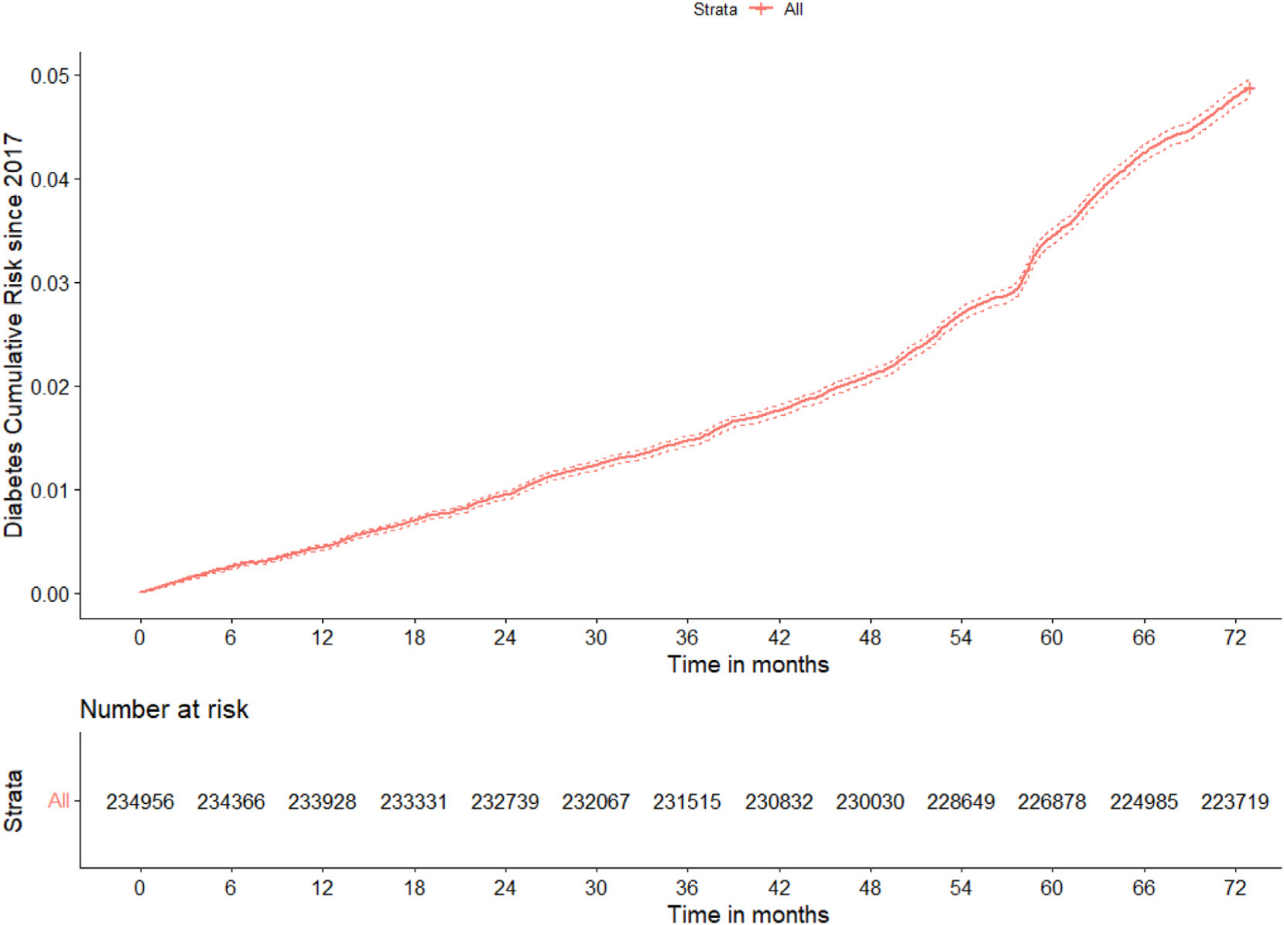
Cumulative risk of new-onset T2D in the 6-year observation period (2017–2022).

The cumulative risk of incident T2D in the 6-year observation period is shown in Fig. 4, unveiling a marked steepening during the COVID-19 pandemic.

The results of regression analysis conducted to investigate the variables that are associated with the risk of T2D over the 6-year observation period are reported in Supplementary Table S1. All the considered predictive factors, except for glutamic oxaloacetic transaminase (GOT) plasma concentration, were significantly associated with the risk of T2D.

### Analyses in patients with prediabetes

To verify whether the increased risk of T2D was present also in individuals who already had prediabetes, we analysed a subgroup of individuals with glucose levels between 100 and 125 mg/dl. In the prediabetic subgroup the incidence rate of new-onset T2D per 1000 person-years increased about once and a half (RR: 1.58) with a 21.78 incidence rate (95% CI, 20.54–23.09) in the years 2017–2019 increasing to 34.41 (95% CI, 32.84–36.05) in the years 2020–2022 (Supplementary Figure S4). The log-linear model estimated monthly growth rate in the pre-pandemic period was 0.003% [95% CI, 0.000–0.015]; the log-linear model estimated monthly growth rate in the pandemic period was 0.023% [95% CI, 0.006–0.040].

**Fig. 5:**
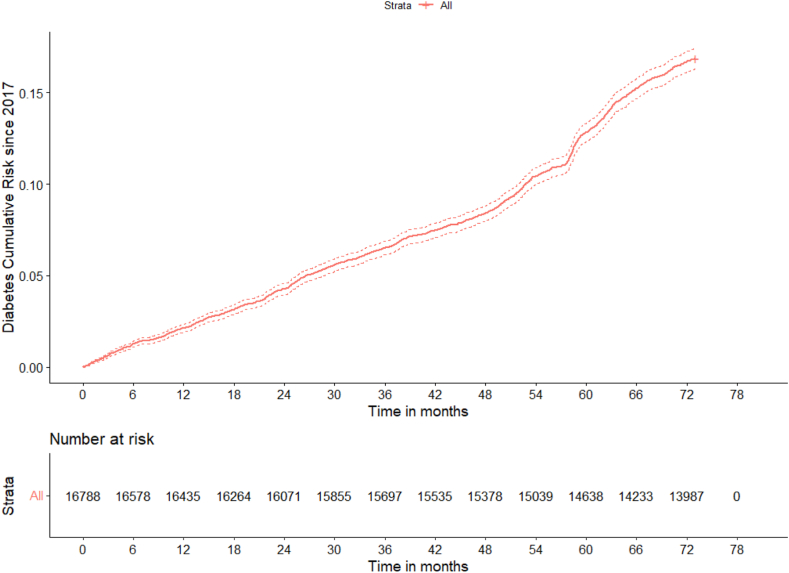
Cumulative risk of T2D in the 6-year observation period in the cohort subgroup with fasting glucose levels between 100 and 125 mg/dl (prediabetes).

The estimated doubling time of the number of new diagnoses of T2D was 228 months in the pre-pandemic period *vs* 30 months in the pandemic period (Fig. 5). Regression analysis reporting the predictive factors of patients with prediabetes over the 6-year observation period are shown in Supplementary Table S2. All the considered predictive factors, except gender and hypercholesterolemia, were significantly associated with the risk of T2D.

## Discussion

To better understand the changing incidence of T2D during the continued course of the COVID-19 pandemic, we compared the incidence of T2D during the pandemic period with its incidence during the pre-pandemic period in a large sample of the general population using a single cohort ITS approach. Our results demonstrate an alarming and progressive increase of new-onset T2D during the 3 years of the COVID-19 pandemic compared with the 3 years preceding the pandemic.

To the best of our knowledge, our study is the first to be performed on a large sample of the general population and not on databases of hospitals or outpatient clinics. Indeed, in Italy all citizens have a primary care physician (family doctor) who follows them even if they do not have specific pathologies. Therefore, the database that we used includes not only patients with known illnesses but also individuals who do not have any disease (or at least are not aware of). This situation currently applies to COVID-19 infection since the attenuation of symptoms and the lack of meticulous tracking of the infection have resulted in many individuals contracting the disease without being aware of it, or not reporting this condition to the health authorities.

A major strength of our study is given by having access to data on the population in the years before the pandemic and during the pandemic; so, instead of constructing two similar samples using propensity score, we used an ITS approach on a single cohort with the only variation being the appearance of the pandemic in the second three-year of follow-up (2020–2022) compared to the first period of observation (2017–1019). When performing the “before *vs* after” comparison, we also considered the time effects, since even without the COVID-19 pandemic, there might still be an increasing trend of T2D incidence over time, especially with a relatively long follow-up period. However, the lack of any difference in the mean age of the two cohorts, the stable incidence during the first three years of follow-up and the sharp increase of the monthly incidence of new T2D during the second half of the observation period make this hypothesis unlikely. In this way, we also managed to overcome the issue of lack of awareness of the disease, which has been a crucial limitation in previous studies due to the impossibility of ensuring that the control group was truly infection-free.

Our rate of incident T2D in the pre-pandemic years is comparable with the one reported by Naveed and colleagues (5.08 per 1000 patient/years),^29^ whilst during the COVID-19 pandemic we detected an incidence that is almost double as compared with the one reported by these investigators, and more similar to the 85% increase described by Xie and Al-Aly^28^ when the outcome was based on use of glucose-lowering medication. Indeed, the increased risk of newly diagnosed diabetes can be particularly evident when the continued use of hypoglycaemic drugs is used as a discriminant for diagnosing this condition.^28^ By employing this type of evaluation, we found that the incidence of new-onset T2D rises during the pandemic period from 4.8 to 12.2 per 1000 person-years. It is also important to note that the screening rate during the lock-down and generally during the pandemic was reduced due to the restrictions to the access to hospitals and outpatient settings.^48^, ^49^, ^50^

In one of the largest and most wide-ranging analyses of this kind, Zhang and colleagues found an incidence of diabetes of 15.53 per 1000 person-years during COVID-19, considering only infected people.^27^ These data seem to uphold the assumption that the true rate of COVID-19 infections in the general population is not fully known.^3^ In fact, it is possible that some of the individuals enrolled might have contracted SARS-CoV-2 without being actually tested for it, and if these people were present in large numbers within the contemporary control group, this event might have biased the results towards the null hypothesis.^28^ Further supporting our findings, an excess of diabetes-related deaths during the COVID-19 pandemic has been recently reported in the United States.^51^

Using Cerner Real-World Data from the US, Qeadan and coworkers^52^ found significantly higher odds of developing new-onset T1D in patients with COVID-19 (odds ratio, 1.42; 95% CI, 1.38–1.46) compared to those without COVID-19. However, that study included participants without any age-based exclusion, whereas our study sample included only adults since in Italy health assistance of younger people is performed exclusively by paediatricians (not included in COMEGEN).

Lastly, we considered only the new occurrence of T2D (not T1D) and only in adults, which represents another strength, as most of the studies available in the literature have focused their attention exclusively on paediatric populations and/or on the risk of developing T1D (unclear if directly caused by SARS-CoV-2 infection of pancreatic beta cells or autoimmune-mediated).^1^^,^^13^^,^^25^^,^^35^^,^^41^^,^^44^^,^^52^, ^53^, ^54^, ^55^, ^56^, ^57^, ^58^, ^59^, ^60^, ^61^, ^62^, ^63^, ^64^

According to recent analyses, the global burden of prediabetes is substantial and growing.^65^, ^66^, ^67^ Our data showing the increased risk of developing T2D in patients with prediabetes support an enhanced prediabetes surveillance, especially after the COVID-19 pandemic, in order to effectively implement diabetes prevention policies and interventions.

Our study is not exempt from limitations. For instance, we do not have data on modifications of lifestyle behaviours including physical activity, dietary habits, and sleeping patterns, which could have played a role in increasing the risk of diabetes after COVID-19^68^, ^69^, ^70^ as well as on vaccination, which has been shown to reduce the severity and long-term effects of COVID-19, including the increase in the onset of new diabetes.^71^, ^72^, ^73^, ^74^ Additionally, although individuals could enter or leave the medical records during the 6-years observation period, artificially small numbers did so within our study cohort due to how we formed it, leading to ageing and/or changing characteristics in the cohort over time. This lack of comparability of the demographic characteristics between the pre-pandemic and the pandemic years could have introduced confounding and selection biases in the results. We acknowledge that our results do not allow any speculation on the pathophysiologic mechanisms underlying the increased rate of incident T2D in the pandemic period, which could include direct effects of SARS-CoV-2 infection, and indirect effects like stress, changes in diet and exercise and in cardiovascular prevention strategies, and access to health system during pandemic; other possibilities include an injury of insulin-secreting pancreatic β cells, inflammation, autoimmune dysregulation, and endothelial dysfunction^75^, ^76^, ^77^, ^78^, ^79^, ^80^; however, defining the exact mechanisms is beyond the scope of this epidemiologic study. Nonetheless, the pivotal role of HbA1c in identifying individuals who were at highest risk of new-onset T2D as assessed by the use of anti-hyperglycaemic drugs seems to support the conclusion that COVID-19 could likely amplify baseline risks and further accelerate manifestations of the disease among individuals already at high risk.

While we do believe that history of COVID-19 positivity has an impact on the risk of T2D, we also trust that the positivity reported in our sample is greatly underestimated compared to the prevalence measured in Italy (∼40%, depending on the Region). In Campania alone (the Region where Naples is located), for example, there have been more than 2.4 millions of cases of COVID-19 in the three years of the pandemic, and the total population of Campania is between 5.5 and 6 millions. Thus, since the proportion of individuals with a history of COVID-19 positivity is largely underreported in our dataset, we decided not to include this information in the model as this strategy would have introduced bias.

In conclusion, the present analysis shows that the incidence of T2D increased significantly during the COVID-19 pandemic in Naples, Italy. This finding was also validated in a cohort of patients with prediabetes. Taken together, our data indicate that COVID-19 is associated with an augmented incidence of T2D in a large cohort of adult individuals. The epidemiology of the disease may vary in terms of rates of outcomes as well as in terms of costs and social burden for public health systems. Therefore, COVID-19 survivors will require dedicated clinical follow-up programs to prevent T2D. In this regard, our findings in individuals with prediabetes are of particular importance, emphasizing how this group needs scrupulous attention on diagnosis and management.

## Contributors

GS and BT contributed to the study design. RI, DP, VT, MVM, AL, RP, PG, GE, ML, GP, and CM provided methodological support. RI, DP, AL, GS, and BT managed the data. All authors contributed to data interpretation. GS acquired funding and provided supervision. The manuscript was written by GS and BT. All authors revised the manuscript for important intellectual content, participated in the decision to submit the manuscript for publication, and approved the final submitted version.

## Data sharing statement

Once the data datasets have been fully de-identified and all the main findings have been published, data collected for this study will be shared upon reasonable request to the last author for research purposes, with a signed data access agreement.

## Declaration of interests

All authors declare no competing interests.

## Appendix A Supplementary data

Supplementary Material

Abstract in Italian
