# Supplementary material for: Incidence of type 2 diabetes before and during the COVID-19 pandemic in Naples, Italy: a longitudinal cohort study
Source: eClinicalMedicine. 2023 Dec 5;66:102345. doi: 10.1016/j.eclinm.2023.102345 (PMC10746394; doi:10.1016/j.eclinm.2023.102345)
Supplement: Supplementary Material [file mmc1.pdf]

Supplementary Material:

Table S1

Table S2

Figure S1

Figure S2

Figure S3

Figure S4

T2D (time-to-event)<br><br>Sample: Whole cohort (N= 234956)			
**Characteristic**	**HR**	**95% CI**	**p**
**Age**	1.05	1.05, 1.05	**<0.001**
**Sex**			
**F**	—	—	
**M**	1.30	1.25, 1.34	**<0.001**
**BMI (Kg/m^2)**	1.12	1.11, 1.12	**<0.001**
**Creatinine (ml/dl)**	6.20	5.27, 7.30	**<0.001**
**Cancer**	1.41	1.35, 1.47	**<0.001**
**COPD**	3.32	3.12, 3.54	**<0.001**
**Hypertension**	6.15	5.90, 6.41	**<0.001**
**Hypercholesterolemia**	2.53	2.43, 2.64	**<0.001**
**Hypertriglyceridemia**	3.92	3.58, 4.30	**<0.001**
**Glycemia (mg/dl)**	1.08	1.08, 1.08	**<0.001**
**HbA1c (mg/dl)**	3.13	3.04, 3.23	**<0.001**
**GPT (U/L)**	1.01	1.01, 1.02	**<0.001**
**GOT (U/L)**	1.00	1.00, 1.01	0.66

Supplementary Table 1. Regression analysis assessing the predictive factors of diabetes in the total 6-year cohort. Significant p-values are marked in bold.

aHR: adjusted hazard ratio; BMI: body mass index; COPD: chronic obstructive pulmonary disease; GOT: glutamic oxaloacetic transaminase; GPT: glutamic pyruvic transaminase; HbA1c: glycated haemoglobin; T2D: type 2 diabetes.

T2D (time-to-event)<br><br>Sample: Prediabetic subjects				
**Characteristic**	**N**	**HR**	**95% CI**	**p**
**Age**	16779	1.02	1.02, 1.02	**<0.001**
**Sex**	16779			
**F**		—	—	
**M**		1.06	0.99, 1.14	0.11
**BMI (Kg/m^2)**	12441	1.04	1.03, 1.05	**<0.001**
**Creatinine (ml/dl)**	7033	1.98	1.50, 2.62	**<0.001**
**Cancer**	16788	0.91	0.84, 0.99	**0.027**
**COPD**	16788	1.49	1.32, 1.68	**<0.001**
**Hypertension**	16788	2.03	1.86, 2.23	**<0.001**
**Hypercholesterolemia**	16788	1.02	0.94, 1.10	0.69
**Hypertriglyceridemia**	16788	1.83	1.56, 2.14	**<0.001**
**Glycaemia (mg/dl)**	16788	1.08	1.08, 1.09	**<0.001**
**HbA1c (mg/dl)**	5845	2.88	2.71, 3.06	**<0.001**
**GPT (U/L)**	11583	0.99	0.99, 1.00	**0.029**
**GOT (U/L)**	11673	0.98	0.97, 0.99	**<0.001**

Supplementary Table 2. Regression analysis reporting the predictive factors of diabetes in the cohort subgroup with fasting glucose levels between 100 and 125 mg/dl (prediabetes). Significant p-values are marked in bold.

aHR: adjusted hazard ratio; BMI: body mass index; COPD: chronic obstructive pulmonary disease; GOT: glutamic oxaloacetic transaminase; GPT: glutamic pyruvic transaminase; HbA1c: glycated haemoglobin; T2D: type 2 diabetes.

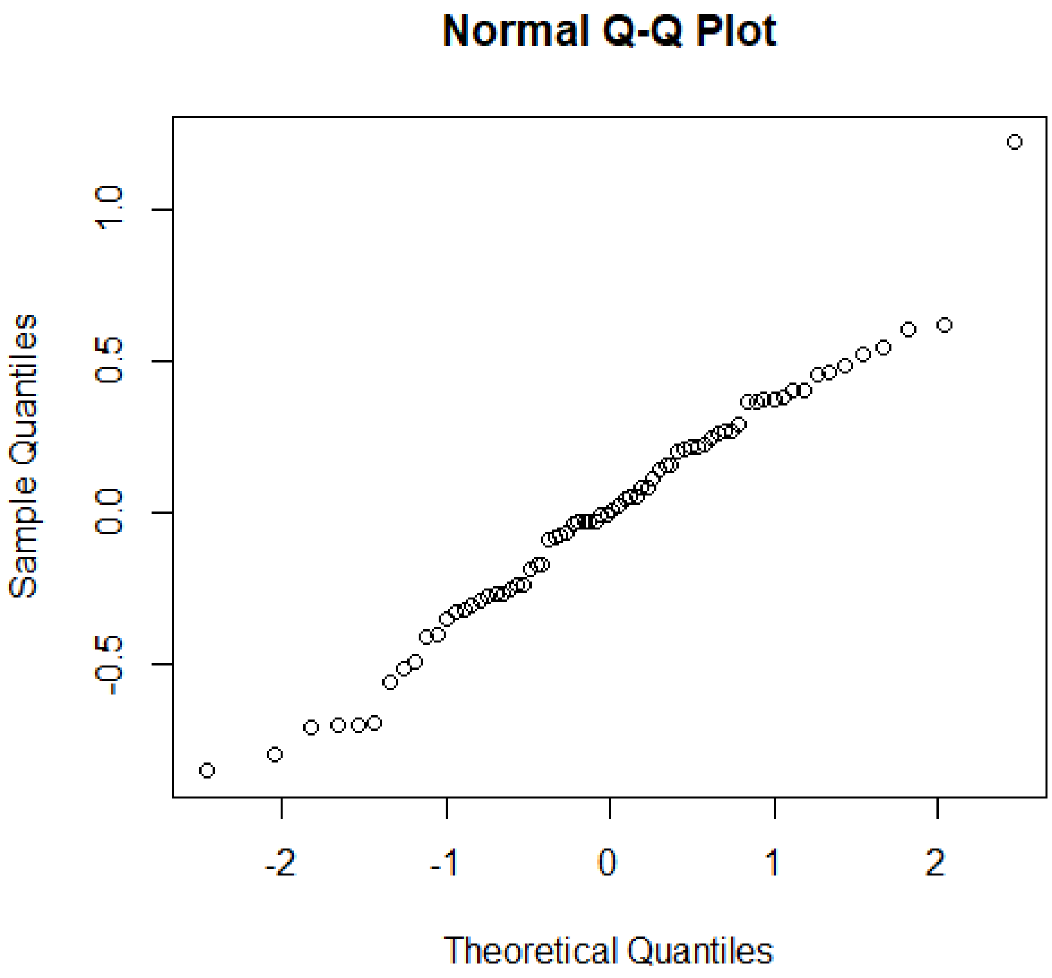

Supplementary Figure 1. Q–Q plot (quantile-quantile plot) of the Poisson regression model used to estimate the incidence trend over time.

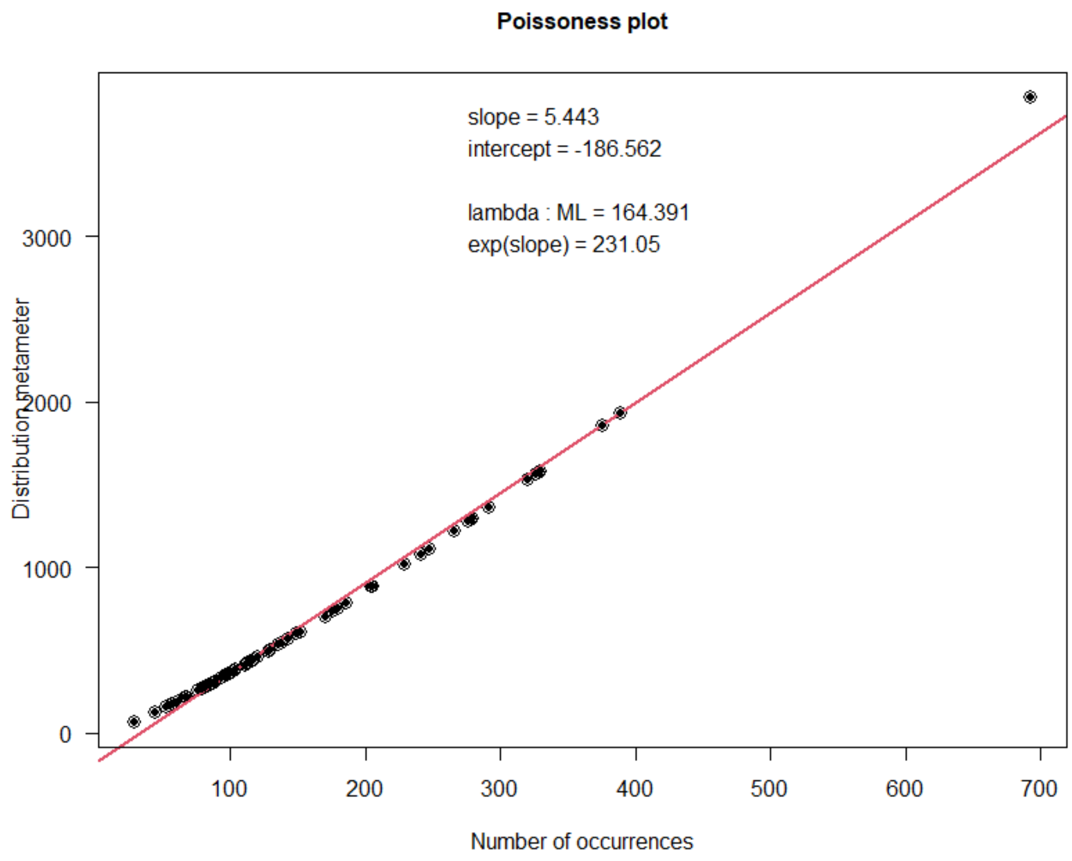

Supplementary Figure 2. Poissoness plot of the Poisson regression model used to estimate the incidence trend over time.

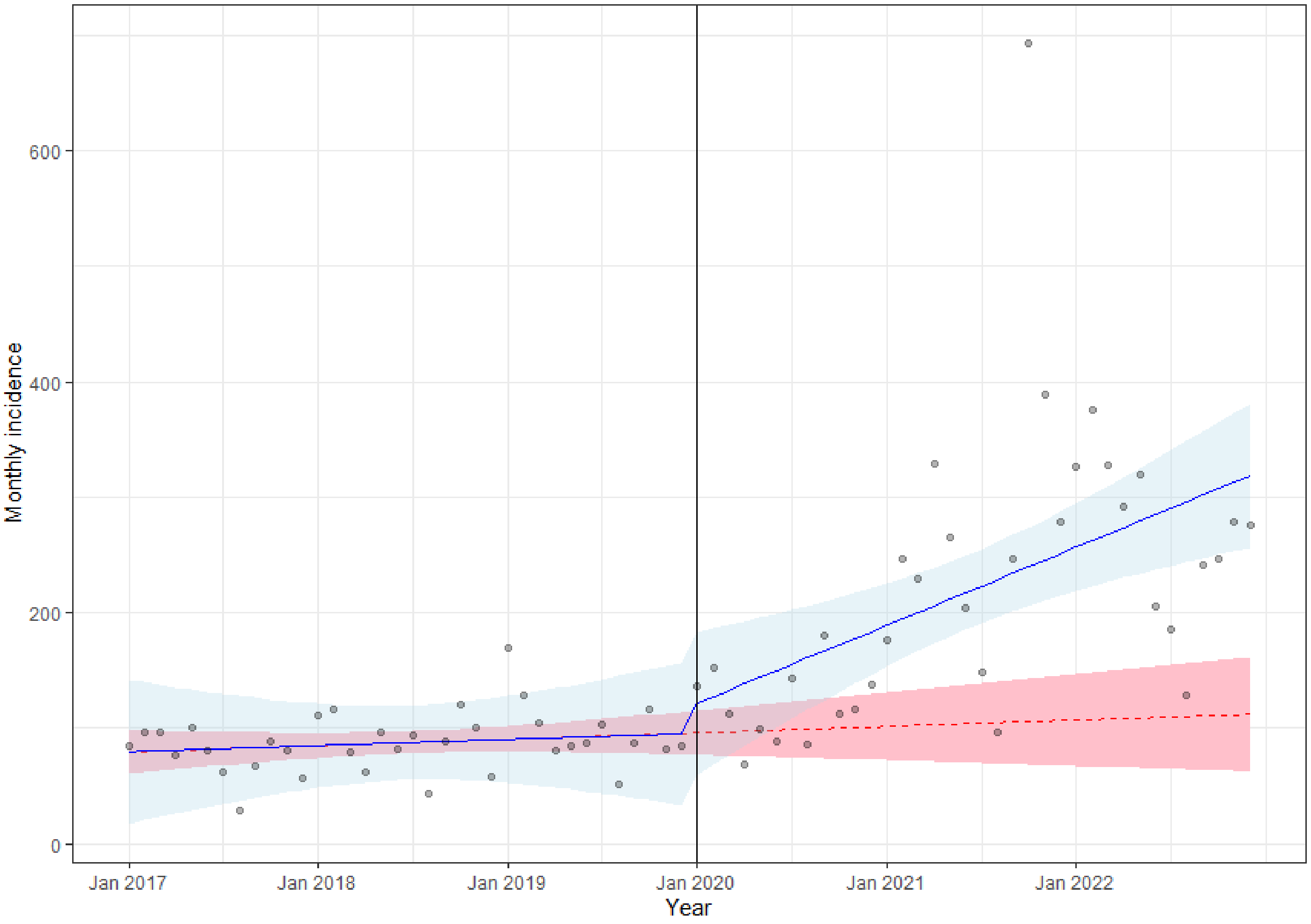

Supplementary Figure 3. Interrupted time series analysis on the monthly incidence of T2D in the 6-year observation period (2017-2022). Red dashed line (with 95% confidence bands) shows counterfactual incidence trend while the blue solid line (with 95% confidence bands) shows the observed incidence trend. Black vertical line marks the beginning of the hypothesised exposure to COVID-19.

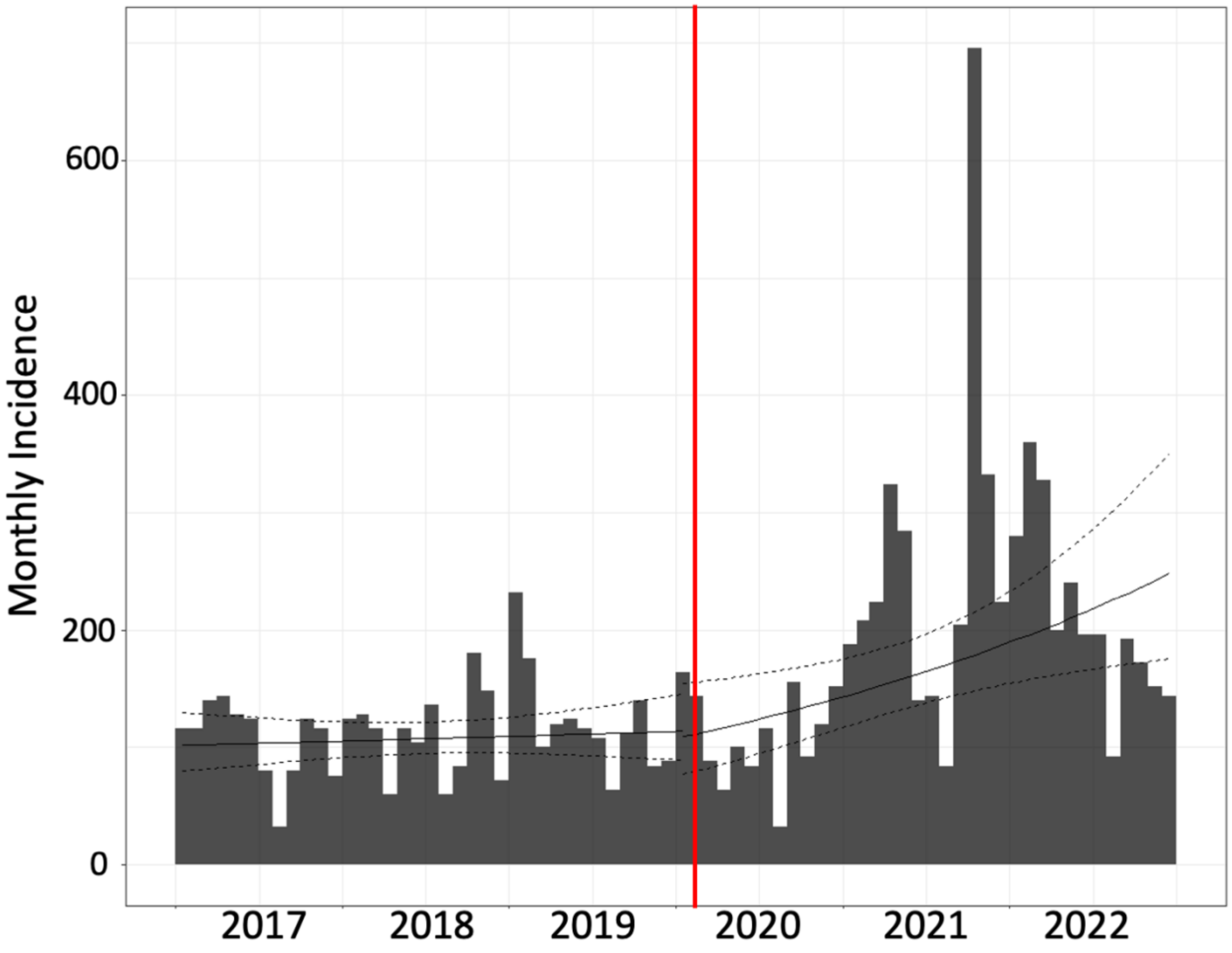

Supplementary Figure 4. Monthly incidence of T2D in the 6-year observation period (2017-2022) in the cohort subgroup with fasting glucose levels between 100 and 125 mg/dl (prediabetes) showing the log-linear regression lines and 95% confidence bands.
