# Supplementary material for: Incidence of type 2 diabetes before and during the COVID-19 pandemic in Naples, Italy: a longitudinal cohort study
Source: eClinicalMedicine. 2023 Dec 5;66:102345. doi: 10.1016/j.eclinm.2023.102345 (PMC10746394; doi:10.1016/j.eclinm.2023.102345)
Supplement: Abstract in Italian [file mmc2.docx]

**Introduzione -** L'associazione tra COVID-19 e lo sviluppo di diabete mellito è stata recentemente investigata da diversi gruppi, producendo risultati controversi. Gli studi attualmente disponibili in letteratura si occupano principalmente di diabete mellito di tipo 1 (DMT1), confrontando pazienti con un test positivo al SARS-CoV-2 con individui senza COVID-19, soprattutto in popolazioni pediatriche. Nel presente studio, abbiamo cercato di determinare l'incidenza di diabete mellito di tipo 2 (DMT2) prima e durante la pandemia.

**Metodi -** In questo studio longitudinale di coorte, abbiamo analizzato una coorte chiusa seguita per un periodo di 6 anni utilizzando un approccio a serie temporali interrotte, ovvero 3 anni prima e 3 anni durante la pandemia. Abbiamo analizzato le cartelle cliniche di oltre 200.000 adulti ottenute da un'associazione di medici di medicina generale a Napoli nel periodo 1 Gennaio 2017 - 31 Dicembre 2022. In questo modo, abbiamo avuto l'opportunità di confrontare l'incidenza di nuovi casi di DMT2 prima (2017-2019) e durante (2020-2022) la pandemia da COVID-19. I principali criteri di inclusione erano età superiore a 18 anni e disponibilità dei dati per il periodo di osservazione; i pazienti con una diagnosi di diabete ottenuta prima del 2017 sono stati esclusi. L'outcome principale dello studio era la numerosità di nuove diagnosi di DMT2, seguendo le definizioni della Classificazione Internazionale delle Malattie 10 (ICD-X), considerando anche la prescrizione di terapie anti-diabete per oltre 30 giorni.

**Risultati -** 234.956 soggetti sono stati seguiti per almeno 3 anni prima o 3 anni durante la pandemia e sono stati inclusi nello studio; tra questi, 216.498 sono stati analizzati negli anni pre-pandemici e 216.422 negli anni pandemici. Il tasso di incidenza di DMT2 è stato di 4,85 (95% C.I. 4,68-5,02) per 1000 persone-anno nel periodo 2017-2019, rispetto a 12,21 (95% C.I. 11,94-12,48) per 1000 persone-anno nel 2020-2022, con un aumento di circa due volte e mezzo. Inoltre, il tempo di raddoppio del numero di nuove diagnosi di DMT2 stimato dal modello di Poisson non adattato è stato di 97,12 (95% C.I. 40,51-153,75) mesi nel periodo pre-pandemico rispetto a 23,13 (95% C.I. 16,02-41,59) mesi durante la pandemia. Questi risultati sono stati confermati anche quando abbiamo esaminato i pazienti con pre-diabete.

**Interpretazione -** I dati del nostro studio di 6 anni che ha coinvolto oltre 200.000 partecipanti adulti indicano che l'incidenza di DMT2 è stata significativamente più alta durante la pandemia rispetto alla fase pre-COVID-19. Di conseguenza, l'epidemiologia della malattia potrebbe cambiare sia in termini di prevalenza che di costi per la sanità pubblica. I sopravvissuti al COVID-19, soprattutto i pazienti con pre-diabete, potrebbero necessitare di programmi clinici specifici per prevenire il DMT2.
